# Retrospective Analysis of Endocrine Dysfunctions in a Population of Adult Polytransfused Patients: Correlation of GH-IGF1 Axis Alteration with Cardiac Performance

**DOI:** 10.1155/2018/6047801

**Published:** 2018-09-26

**Authors:** Michela Rosaria Campo, Anna Farese, Michele Correale, Giuseppe Berti, Michela Massa, Maria Rosaria Sorrentino, Grazia Roberti, Filomena Sportelli, Mauro Cignarelli, Olga Lamacchia

**Affiliations:** ^1^Endocrinology and Metabolic Diseases Unit, Department of Surgical and Medical Sciences, University of Foggia, Via Luigi Pinto 1, Foggia, Italy; ^2^Cardiology Unit, Department of Surgical and Medical Sciences, University of Foggia, Via Luigi Pinto 1, Foggia, Italy; ^3^Endocrinology Unit, Scientific Institute Casa Sollievo della Sofferenza, San Giovanni Rotondo, Viale Cappuccini 1, Foggia, Italy; ^4^Transfusional Medicine Unit, Azienda Ospedaliero Universitaria Ospedali Riuniti, Via Luigi Pinto 1, Foggia, Italy

## Abstract

Endocrine complications of haemochromatosis and heart failure mostly affect morbidity and mortality in polytransfused patients. This study analyzes endocrine dysfunctions and the impact of GH-IGF-1 axis alteration on cardiac performance in a population of 31 patients. A retrospective study on 31 Caucasian polytransfused outpatients, 27 adults and 4 pediatric, residing in Apulia, Italy, followed from 2005 to 2016, was conducted. Patients underwent basal and dynamic hormonal evaluation. GHRH plus arginine test was performed in 21 patients (19 adults and 2 children). Among them, 9 patients were affected by left ventricle diastolic dysfunction and/or atrial or ventricular dilatation (HD group) and 12 patients did not have cardiovascular disease (non-HD group). Twenty-nine out of 31 patients (94%) had at least one endocrinopathy. We found severe or mild GH deficit (GHD) in all HD patients versus 3 patients in the non-HD group (p=0.001). Mean IGF-1 levels were significantly lower in the HD group than in non-HD subjects (53±30 versus 122±91 *μ*g/L, p=0.04). Our study confirms the need to perform a dynamic evaluation of the GH-IGF1 axis in polytransfused patients, especially when heart dysfunction emerges. An intervention study with GH replacement therapy in a larger randomized adult population will clarify the role of GH/IGF axis on cardiovascular outcomes in this patient population.

## 1. Introduction

Despite the availability of effective new iron-chelating drugs, secondary haemochromatosis due to iron deposition in endocrine glands remains the main factor contributing to the endocrine complications in patients treated with periodic transfusional regimen because of *β*-thalassaemia and other haemoglobinopathies. Hypogonadotropic hypogonadism (HH), delayed puberty, and GH defect (GHD) are indeed the most frequent endocrine alterations in such patients [1, 2] and they also contribute to osteopenia and osteoporosis, present in over 50% of patients [3]. Studies regarding the function of growth hormone (GH) and insulin like growth factor 1 (IGF-1) axis have shown a GHD in about 30% of polytransfused patients [2, 4, 5] and reported that lower IGF-I levels are correlated with a worst bone mineral density (BMD) [2, 5–7]. Unfortunately, the true meaning of lower IGF-I levels in these patients is unclear, especially in older patients, due to nutritional disturbances and to concomitant chronic HCV correlated hepatopathy, which, though less frequent in recent years, affects the diagnosis and therapeutical response to GHD correction [8, 9]. In addition to endocrine dysfunctions, dilated cardiopathy (DC) and chronic heart failure (CHF) are the pathologies that most exert a negative influence on quality of life, morbidity, and mortality in adult polytransfused patients [4]. Among several possible causes, impaired GH/IGF-I axis may contribute to cardiovascular outcomes in both polytransfused [2, 10] and in nonpolytransfused patients [11], but in the literature, there is a lack of clinical data on this issue. The purpose of this observational study was to explore GH-IGF-1 axis function in a population of mainly adult polytransfused patients and correlate it with cardiovascular function.

## 2. Materials and Methods

This retrospective study was conducted in 31 (M/F: 17/14) Caucasian patients, resident in the north-eastern part of Apulia, in south-eastern Italy, who were regularly transfused at our Transfusional Center, whom we followed from 2005 to 2016 at the Endocrinology and Diabetology Unit, University of Foggia. The cohort at baseline included 27 adults (median age 42 years, range 23-62) and 4 pediatric patients (median age 11.5 years, range 9-13) but the long-term follow-up finally produced an adult population analysis. Twenty-four patients were affected by *β*-thalassemia major (TM), six by thalassemia intermedia (TI), one by drepanocytosis and dyserythropoietic anaemia. Most had been regularly transfused since early childhood and had been undergoing chelation therapy with the use of desferrioxamine replaced by deferasirox for the past 7-9 years. One 39-year-old patient affected by TM had undergone successful medullary transplantation when he was 23. One 39-year-old patient affected by TI had undergone regular transfusions between the ages of 15 and 29, while one TI patient had begun transfusional regimen at 60 years of age.

At the time of the study, Hb levels ranged from 9.5 g/dl to 11.2 g/dl. Patients underwent periodic (3-12 months) clinical examination, routine laboratory tests, and dynamic tests for metabolic and hormonal evaluation. Twenty-two patients were positive to HCV, of whom 12 had chronic liver disease and 5 had been undergoing interferon/ribavirin/sofosbuvir therapy, finally all 22 achieved undetectable HCV-RNA levels. In all patients, blood samples were drawn at 8.00 AM after an overnight fast and at least 2 weeks after the previous blood transfusion in order to measure the serum concentrations of urea, creatinine, electrolytes, glucose, calcium, phosphate, alanine aminotransferase (ALT), gamma glutamyltransferase (*γ*GT), alkaline phosphatase (ALP), total and direct bilirubin, albumin, prothrombin time (PT) and international normalization ratio (INR), 1-25 OH vitamin D (23 patients), TSH, FT3, FT4, and cortisol. Periodic serologic screening assays for HCV (HCVab and HCV-RNA), HBV, and HIV seropositivity were also performed.

Serum HCV-RNA levels were measured using the Abbott Real Time HCV assay, with a lower limit of quantitation and detection of 12 IU/mL. The lower limit of detection was used in the determination of undetectable HCV-RNA. Testosterone and PRL levels were measured annually in males. PRL, LH, and FSH concentrations were measured in female patients with oligo-amenorrhoea. One patient underwent an OGTT test, two patients a standard-dose corticotropin test, and 21 a GHRH plus arginine test (19 adults and 2 children, m/f 9/8, mean age 35.5±11, BMI 21.7±2.6). All the tests were performed in compliant patients within the first twelve months from the first evaluation at our Centre independently of the presence of cardiological disease. Among the 21 patients who underwent GHRH test, 9 patients were affected by variable heart diastolic dysfunction grade, atrial or ventricular dilatation with a history of arrhythmias (HD patients, mean age 35.5±10.4 years), while 12 patients did not report any history of cardiovascular disease (non-HD patients, mean age 32.6±10 years). We repeated GH reserve testing in 5 patients at variable intervals (1-6 years) because of worsening of asthenia and/or a previous partial GHD or due to onset of cardiac complications. One of these retested patients underwent an insulin tolerance test (ITT) instead of the GHRH test due to a suspicion of associated cortisol deficiency. Thirteen tested patients were affected by HH (9 males and 4 females) and were on stable sex steroid replacement for at least one year before GHRH testing.

*Test Procedures*. GHRH plus arginine: an indwelling catheter was placed into a forearm vein between 07:00 and 08:00 AM and continuously flushed with saline. Blood samples for GH evaluation were collected 15 and 0 min prior to and 30, 60, and 90 min after the stimulation test (GHRH1-29; GEREF, Serono, Italy; 1* μ*g/kg i.v. at 0* *min; ARG hydrochloride, 0.5* *g/kg over 30* *min from 0* * to+30* *min, up to a maximum of 30* *g). According to the literature [12], a GH peak response>11 *μ*g/L was considered normal for those with a body mass index (BMI) <25 kg/m^2^; a GH peak >8 *μ*g/L for BMI 25–30 kg/m^2^; a GH peak >4 *μ*g/L for BMI >30 kg/m^2^. A threshold of 16 *μ*g/L was used to define mild GHD [12].

ITT: an indwelling catheter was placed into a forearm vein between 07:00 and 08:00 AM and continuously flushed with saline. Injection of 0.15 IU/kg regular insulin (Actrapid, Novo Nordisk) was performed in order to achieve blood glucose levels less than 40 mg/dl and until symptoms of hypoglycemia developed. Blood samples for cortisol and GH evaluation were taken at 0, 30, 45, 60, and 90 min. A threshold of 500 nmol/L of cortisol and 3 *μ*g/L of GH was used to define adrenal insufficiency and severe GHD, respectively [12, 13].

Corticotropin test: Exogenous 1–24 ACTH (Synacthen 0,25 mg/mL, Novartis Pharma) was administered intramuscularly and a blood sample for cortisol evaluation was taken 60 min after stimulation. A threshold of 500 nmol/L was used to define adrenal insufficiency [13].

GH serum concentrations were measured by a two-site chemiluminescent immunoassay (Immunolite 2000; DPC, Diagnostic Products Corporation, Los Angeles CA). The limit of detection was 0.01 *μ*g/L with intra- and interassay variation coefficients of 3.5% and 6.5%, respectively. IGF-1 concentrations were determined by a two-site chemiluminescent immunoassay (Liaison® DiaSorin) after acid-ethanol extraction. The sensitivity of the method was <3 *μ*g/L. The intra and interassay variation coefficients were 8 and 12%, respectively. IGF-1 concentrations were expressed as absolute values and referred to age. The other hormonal determinations were performed by commercially available chemiluminescence immunoassays kits. The intra- and interassay variation coefficient for all methods were <5.8% and 10.8 %, respectively. Serum ferritin was measured by electrochemiluminescent immunoassay (Ortho Clinical Diagnostics, Johnson & Johnson Medical S.p.A). Reference range was 25-380 *μ*g/L in males and 10-200 *μ*g/L in females. Thyroid ultrasound and conventional 2D and tissue velocity imaging (TDI) echocardiography in the ambulatory setting and under resting conditions were also carried out in all subjects. Bone lumbar and femoral densitometry (DEXA) was performed annually in all patients from the age of 10 years.

Conventional echocardiography was used to assess left ventricle (LV) dimensions and ejection fraction (LVEF), peak velocities of transmitral early (E) and late diastolic (A), LV filling, and the ratio of transmitral early to late (E/A ratio). TDI measurements recorded at the septal mitral annulus in apical four-chamber view included systolic velocity (S'), early (E'), and late (A') diastolic velocities. The transmitral to mitral annular early diastolic velocity ratio (E/E') was also calculated. Transthoracic echocardiography was performed with the use of iE33 (Philips Medical Systems, Andover, MA, USA). All echocardiographic studies were performed and interpreted by experienced physicians, blinded to the GH-IGF-1 data. LV dimensions and LVEF were calculated as recommended in the joint ASE/ESC guidelines. LVEF was calculated according to Simpson's rule. Pulsed Doppler mitral inflow velocities were obtained by placing a 1-2 mm sample volume between the tips of the mitral leaflets in the apical four-chamber view. The Doppler beam was aligned parallel to the direction of flow. In order to detect left ventricle diastolic dysfunction, conventional Doppler and TDI were used, in accordance with previous reports [14, 15].

Osteoporosis was defined by a T score of below -2.5 SD and osteopenia as a T score of between -1 and -2.5 SD [7].

## 3. Statistical Analysis

Data are given as mean value±SD; categorical variables are described as frequencies and percentages. The differences between the two groups were determined using Fisher's exact test for categorical variables and Student's t-test for unpaired data for continuous variables. Alternatively, analysis of variance (ANOVA) was used for comparison of continuous variables between three or more groups. Bonferroni post hoc tests were used to determine significant differences. Statistical analyses were performed using SPSS version 13.0 (SPSS Inc., Chicago, IL, USA). A P-value <0.05 was considered to be significant.

## 4. Results

**Figure 1:**
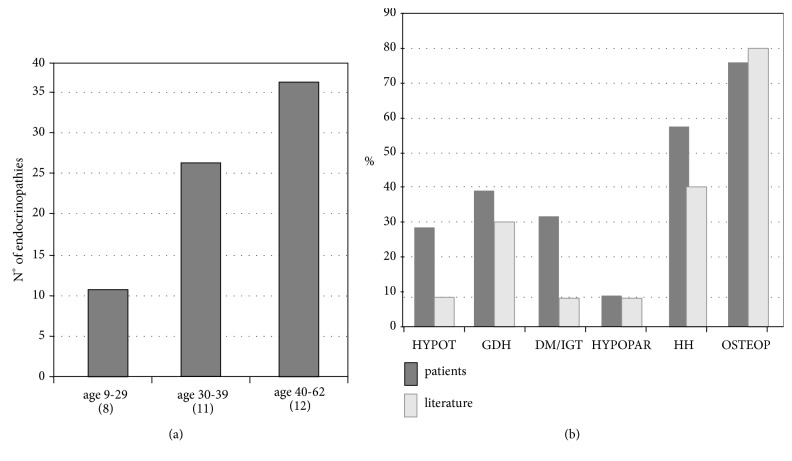
(a) Number of endocrinopathies in 31 polytransfused patients divided into three groups by age. () indicates the number of patients for each group. (b) Frequency of endocrinopathies found in 31 polytransfused patients and comparison with literature. Hypot: primary hypothyroidism; GHD: severe+mild GH deficiency; DM/IGT: diabetes mellitus or impaired glucose tolerance; Hypoparat: primary hypoparathyroidism; HH: hypogonadotropic hypogonadism; Osteop: osteoporosis or osteopenia.

At the time of the study, serum ferritin (mean ± SD; *μ*g/L) in males was 1125±1093 and 1036±777 *μ*g/L in female. Anthropometric and ferritin data are reported in Table 1. Twenty-nine out of 31 patients (94%) had at least one endocrinopathy. As expected, patients with one or more endocrinopathies were older (p=0.02) (Figure 1(a)). In our study, recent serum ferritin was correlated to the number of endocrinopathies (p=0.04).

The alterations in endocrine functions are summarized in Figure 1(b). In our study, 12 males and 6 females showed HH (58%) and were treated with sex steroid replacement with variable compliance. Ten patients (32%) were affected by diabetes mellitus (DM), impaired fasting glucose (IFG), or impaired glucose tolerance (IGT) and treated with diet or insulin (n=5). Primary hypoparathyroidism was diagnosed in 3 patients (10%) and treated with calcium and calcitriol. Primary hypothyroidism was found in 9 patients (29%). All patients presented morning (08.00 AM) cortisol concentrations above 100 nmol/L. Among them, three subjects with cortisol levels between 100 and 550 nmol/L underwent an ACTH test (two subjects, basal cortisol 250 and 400 nmol/L, peak 905 and 1050 nmol/L, respectively) and an ITT test (one subject, basal cortisol 350 nmol/L, peak 885 nmol/L at 60 minutes; fasting basal glucose level 96 mg/dl, nadir at 30 minutes: 39 mg/dl). A GHD was found in 12 adult patients (39%) which was severe in 9 (29%) and mild in 3 patients (9.6%). The mean values of GH during GHRH + arginine test at different time points are reported in Table 3. Mean 1-25 OH vitamin D3 concentrations were 17.4pg/ml±7.

DEXA analysis showed osteoporosis in 16 patients (55%) and osteopenia in 6 subjects (10%).

Echocardiographic examination revealed the presence of valvular insufficiency, mainly of the mitral valve, in 9 patients. Twelve patients (35%) were affected by diastolic dysfunction, ventricular and/or atrial dilatation, history of arrhythmia, and CHF and all were on stable ACE inhibitor or diuretic or beta-blocker treatment.

In the subgroup of patients with structural or functional cardiac anomalies (Heart Dysfunction: HD), the prevalence of DM/IFG was not different as compared with patients with normal cardiac function (data not shown) and we found no HbA1c correlation with serum ferritin, nor with IGF-1 levels.

We divided patients who underwent GHRH testing into those with diastolic dysfunction, atrial and ventricular dilatation, and history of arrhythmia (HD group, 9 patients, mean age 35.5±10.4 years, and BMI 22.4±2.5 Kg/m^2^) and patients without structural and functional cardiac anomalies by echocardiography (non-HD, 12 patients, mean age 32.6±12 years, and BMI 22±3.04 Kg/m^2^) (Table 2). Left ventricular diastolic diameter (LVDD), left atrial diameter (LAD), and E/E' differed significantly in the HD group as compared to non-HD patients. But there were no differences in LVEF and E/A ratio (Table 2).

**Figure 2:**
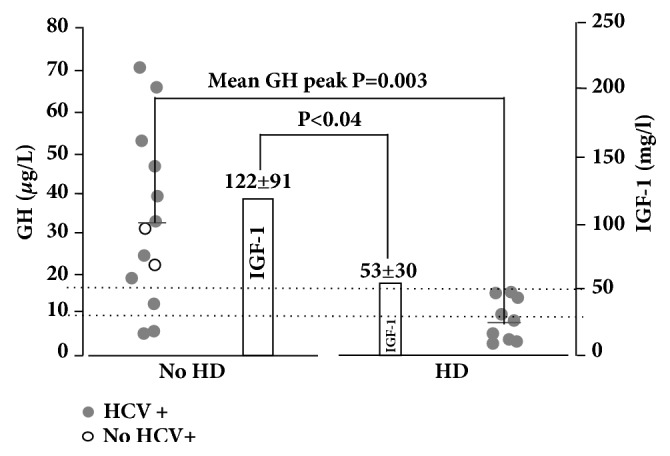
Mean GH peak and IGF-1 mean levels in 21/31 patients (19 adults, 2 children) underwent GHRH plus arginine test. The lines indicate thresholds for severe and mild GHD. Data are shown as mean ± SD.

**Figure 3:**
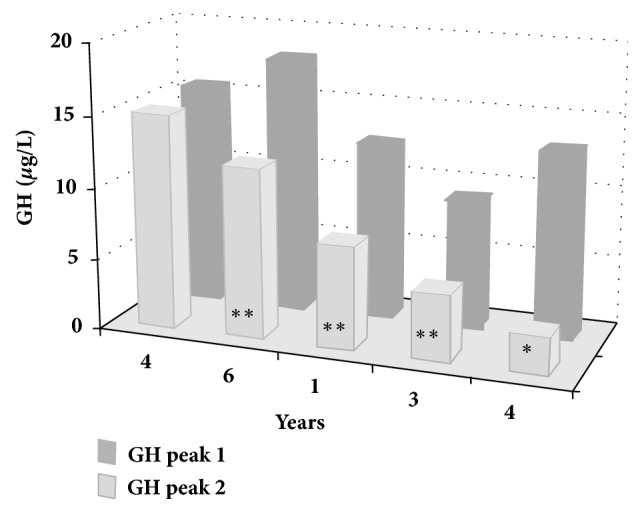
GH peak in 5 patients who underwent repetition of GHRH + arginine testing. Years indicate the interval between tests. *∗* indicates ITT test. *∗∗* indicates retesting for onset of cardiac complications.

The percentage of HCV seropositivity did not significantly differ between the two groups (77 versus 100%, p=0.31) and no patient displayed detectable HCV-RNA levels at the time of the test. GOT-GPT alterations expressed as multiple of upper normal levels correlated to IGF-1 levels but not with GH peak after stimulus. A severe or partial GHD was found in all HD patients (100%) and in 3 from the non-HD group (25%) (p=0.001), with mean GH peak levels of 8.00±5.64 *μ*g/L versus 32.1±21 *μ*g/L, respectively (p=0.003) (Figure 2, Table 2). Mean IGF-1 levels were significantly lower in HD than in non-HD subjects (53±30 *μ*g/L versus 122±91 *μ*g/L, p=0.04), regardless of age, which was not different between the two groups (Figure 2, Table 2). HD patients had a higher number of endocrinopathies versus non-HD patients (3.7 ± 1.2 versus 2.0 ±1.2, p=0.006, Table 2). Among the 5 patients who underwent GH-IGF-1 axis retesting, we found a significant worsening of GH reserve in 4 subjects (Figure 3) and, among them, three patients had developed cardiac complications. Two of the HD patients died, brothers, one aged 24 from ventricular arrhythmia, the other aged 51 from peritoneal fibrosarcoma. No pediatric patient showed impaired GH response to stimulus. We found no correlation between ferritin levels and IGF-1 levels, nor with GH peak after stimulus. We started GH replacement therapy in 5 compliant patients (4 HD and 1 non-HD patients) at variable dosages (0.3-0.8 mg/day). IGF-1 measurements at 12 months showed a consistent mean increase from 46.5±31*μ*g/L to 134.3±86 *μ*g/L and three patients referred improvement of asthenia and stress tolerance. No significant variations in E/E' or LVEF were found in these patients after 12 months of therapy (data not shown).

## 5. Discussion

In our series of patients, we found a prevalence of endocrinopathies similar to that reported in the literature [1–3], except for the diagnosis of DM and IFG/IGT. This discrepancy is probably influenced by the new adopted criteria for DM and IFG management [16]. Older patients showed a higher number of endocrinopathies and this is correlated with the progressive iron accumulation and oxidative damage in endocrine glands [1]. Indeed, we found a significant correlation between ferritin levels and the number of endocrine dysfunctions.

We found left ventricle diastolic dysfunction and ventricular and/or atrial dilatation, with signs or symptoms of heart failure and history of arrhythmia in 35% of our patients, in line with previous studies [17]. The frequency of cardiac function impairment in polytransfused patients is increasing in line with the longer life expectancy by virtue of efficient current chelating therapies that have changed the classical clinical disease course. CHF significantly deteriorates the quality of life and influences the morbidity and mortality of these patients [18]. In a large cohort of TM patients, Pepe et al. [19] demonstrated a significantly higher frequency and a significantly higher risk of cardiac complications (heart failure, hyperkinetic arrhythmias, and myocardial fibrosis) in DM patients versus non-DM patients, but this correlation was not found in our cohort of HD patients which on the other hand showed a higher number of other endocrinological complications.

In accordance with the literature, we found mild or severe GHD in 34% of patients, even though we applied the new proposed BMI correlated cut-off values. GHD is not uncommon in adult polytransfused patients [2, 4, 5] but the frequent coexistence of hepatopathy makes it impossible to use IGF-1 measurement to screen for GHD, so a dynamic test is mandatory, especially in older patients. GHD pathogenesis in polytransfused patients has been classically attributed to iron accumulation and free radical damage to the pituitary gland, albeit a clear correlation between ferritin levels and pituitary damage has not been demonstrated.

In our cohort of adult patients, we found a strong association of GHD with the presence of structural and/or functional cardiac dysfunction. Furthermore, when we repeated GH reserve testing, we found the onset of severe or mild GHD in four out of five subjects retested and among them three patients developed cardiac complications. Cardiac abnormalities are a consequence of the general comorbid conditions in thalassaemia but they may be closely related to concomitant endocrine deficiencies, hypercoagulability state, and inflammatory milieux [18, 19]. Erfurth et al. reported a normalization of cardiac function after GH treatment in a 21-year-old thalassemic female patient who developed end-stage heart failure, thus suggesting a contribution of GHD to heart deterioration [10].

It is well known that the GH/IGF-1 axis regulates cardiac growth, stimulates myocardial contractility, and influences the vascular system [20, 21]. Patients with either childhood- or adulthood-onset GHD develop cardiovascular abnormalities such as reduced cardiac mass, diastolic filling and left ventricular response at peak exercise, increased intima-media thickness, and endothelial dysfunction, reversed, at least partially, by GH replacement therapy [22–25].

In the general population, CHF is a complex syndrome associated with multiple endocrine alterations, including low IGF-1 levels, GHD, and a condition of GH resistance that may be related to the severity of heart disease [11, 26, 27] and the pathogenesis of these alterations is still being debated. In recent studies, worse cardiac function, physical performance, and outcome were reported in CHF associated with GH deficiency [11] and patients with CHF secondary to both ischemic and idiopathic dilated cardiomyopathy displayed significant benefits from GH therapy [28]. Whether GH replacement will finally find a place in the treatment of heart failure, and in what way, remains to be established.

The strong correlation of GHD with the presence of HD found in our population of polytransfused patients should prompt clinical investigators to examine the endocrinological influence and especially the GH-IGF-1 axis abnormalities, on altered heart function and impaired ventricular contractility in such patients, and to analyze the effect of GH replacement therapy on cardiac performance. In our small group of patients treated with GH replacement therapy, despite the coexistence of HCV seropositivity, we found a significant increase in IGF-1 levels and a reported subjective improvement of asthenia and stress tolerance.

This study presents some limitations. The retrospective nature of the study and the small number of analyzed patients limits statistical significance and data interpretation.

## 6. Conclusions

In conclusion, our study suggests that a dynamic test to investigate the GH-IGF1 axis should be mandatory in adult-age polytransfused patients, especially when cardiac dysfunction is manifested. Indeed, we found a strong correlation of GHD with the presence of HD and to our knowledge, this is the first demonstration of such a correlation in adult polytransfused patients. In these patients, an intervention study with replacement GH therapy in a larger randomized population will clarify the influence of GH substitution therapy on cardiac performance, quality of life, morbidity, and mortality.

## Figures and Tables

**Table 1:** Anthropometric, clinical, and ferritin data of 31 polytransfused patients.

**PARAMETER**	**MALE (17)**	**FEMALE (14)**
Mean age (years)	48.5±11	38±13
	(Range 12-62)	(Range 9-48)

Height (m)	1.71±0.10	1.58±0.13
	(Range 146-178)	(Range 1.16-1.68)

BMI (Kg/m^2^)	19.2±3	23.9±5.3
	(Range 15-26)	(Range 17-38)

Ferritin (*μ*g/L)	1125±1093	1036±777
	(Range 127-3540)	(Range 115-3030)

**Table 2:** Anthropometric, laboratory, and echocardiograph data of 21/31 patients who underwent GHRH testing divided according to heart dysfunction (HD).

**PARAMETER**	**HD (n=9)**	**Non-HD (n=12)**	**P**
Mean age (years)	35.5 ± 10.4	32.6±12	NS
(Range)	(23-49)	(9-41)	

BMI (Kg/m^2^)	22.4±2.5	22±3.04	NS

Ferritin levels (*μ*g/L)	1326±1175	1304.6±1048.9	NS

IGF-1 (*μ*g/L)	53±30	122±91	0.04

Mean GH peak (*μ*g/L)	8.00±5.64	32.1±21	0.003

HCV positivity (%)	100	77	NS

Number of Endocrinopathies	3.7 ± 1.2	2.0 ± 1.2	0.006
HH	6	8	NS
DM/IGT	4	2	NS
Hypot	3	2	NS
Hypopar	2	0	NS
GHD	9	3	0.001
Osteop	9	8	NS

LVDD (mm)	52.5±4.7	47.6±4.8	0.03

LAD (mm)	42.25±2	37.7±5.7	0.03

E/E' ratio	11.32±1.6	9.3±1.27	0.04

LVEF (%)	57.75±2.49	60.3±5.53	0.16

E/A ratio	1.92±0.2	1.98±0.35	0.7

HD: heart dysfunction; non-HD: no heart dysfunction; LVDD: left ventricular diastolic diameter; LAD: left atrial diameter; E/E': transmitral to mitral annular early diastolic velocity ratio; LVEF: left ventricular ejection fraction; E/A: ratio of transmitral early to late. Hypot: primary hypothyroidism; GHD: severe+mild GH deficiency; DM/IGT: diabetes mellitus or impaired glucose tolerance; Hypoparat: primary hypoparathyroidism; HH: hypogonadotropic hypogonadism; Osteop: osteoporosis or osteopenia. Data are shown as mean ± SD.

**Table 3:** Mean GH values at different time points during GHRH + arginine test in 21 thalassemic patients.

**TIME (MIN)**	**0**	**30**	**60**	**90**
All patients N 21	1.41±1.6	20.77±17.87	14.61±17.45	5.67±6.5

Severe GHD N 9	0.28 ±0.29	5.18±3.43	2.45±2.11	1.39±1.18

Partial GDH N 3	1.55±1.42	14.83±1.76	6.73±3.45	2.53±1.1

Normal GH reserve N 9	2.27±1.99	34.66±17.74	28.7±18.89	12.61±7.79

GH is expressed as *μ*g/L. Data are shown as mean± SD.

## Data Availability

The data used to support the findings of this study are included within the article.
